# Poor nutritional status of schoolchildren in urban and peri-urban areas of Ouagadougou (Burkina Faso)

**DOI:** 10.1186/1475-2891-10-34

**Published:** 2011-04-19

**Authors:** Charles Daboné, Hélène F Delisle, Olivier Receveur

**Affiliations:** 1 TRANSNUT- Department of Nutrition, Faculty of Medicine, University of Montreal, 2405 Chemin de la Côte Ste Catherine, Montreal Qc, H3T 1A8, Canada; 2 Laboratoire National de Santé Publique, 09 BP 24 Ouagadougou 09, Burkina Faso

## Abstract

**Background:**

Malnutrition is still highly prevalent in developing countries. Schoolchildren may also be at high nutritional risk, not only under-five children. However, their nutritional status is poorly documented, particularly in urban areas. The paucity of information hinders the development of relevant nutrition programs for schoolchildren. The aim of this study carried out in Ouagadougou was to assess the nutritional status of schoolchildren attending public and private schools.

**Methods:**

The study was carried out to provide baseline data for the implementation and evaluation of the Nutrition Friendly School Initiative of WHO. Six intervention schools and six matched control schools were selected and a sample of 649 schoolchildren (48% boys) aged 7-14 years old from 8 public and 4 private schools were studied. Anthropometric and haemoglobin measurements, along with thyroid palpation, were performed. Serum retinol was measured in a random sub-sample of children (N = 173). WHO criteria were used to assess nutritional status. Chi square and independent t-test were used for proportions and mean comparisons between groups.

**Results:**

Mean age of the children (48% boys) was 11.5 ± 1.2 years. Micronutrient malnutrition was highly prevalent, with 38.7% low serum retinol and 40.4% anaemia. The prevalence of stunting was 8.8% and that of thinness, 13.7%. The prevalence of anaemia (p = 0.001) and vitamin A deficiency (p < 0.001) was significantly higher in public than private schools. Goitre was not detected. Overweight/obesity was low (2.3%) and affected significantly more children in private schools (p = 0.009) and younger children (7-9 y) (p < 0.05). Thinness and stunting were significantly higher in peri-urban compared to urban schools (p < 0.05 and p = 0.004 respectively). Almost 15% of the children presented at least two nutritional deficiencies.

**Conclusion:**

This study shows that malnutrition and micronutrient deficiencies are also widely prevalent in schoolchildren in cities, and it underlines the need for nutrition interventions to target them.

## Background

Despite the economic growth observed in developing countries, malnutrition and particularly undernutrition is still highly prevalent [1]. Concurrently, a growing prevalence of obesity and its related chronic diseases is being observed in these countries [2]. Increasing obesity is already a major concern in developed countries for pre-school children [3] as well as schoolchildren[4]. In developing countries, this rising epidemic along with the persistence of undernutrition and infections typifies the 'Double Burden of Malnutrition' (DBM) [5], which is becoming of great concern for African countries [6]. Indeed, the DBM is a real threat at the population, household and even individual level [7], and it is now observed among schoolchildren [8]. Rural areas of developing countries are generally prioritized as regards nutrition intervention, because undernutrition is more widespread than in urban areas [9]. However, a shift is occurring and children in the cities are at risk of both over-nutrition and undernutrition [10]. Some studies are now highlighting the problem of micronutrient deficiencies in cities [11] and among schoolchildren in particular [12]. Schoolchildren are dramatically affected by anaemia [12], vitamin A deficiency [13] and parasitic infections [14] with adverse impact on their nutritional status [15,16], as well as on their cognitive development and school performance [17-19]. Unfortunately, the paucity of nutrition information on this vulnerable population makes it difficult to define appropriate intervention strategies. Demographic and Health Surveys (DHS), which provide nutritional status data at national level, do not include schoolchildren [11,20-23]. Furthermore, the few available data usually pertain to rural schoolchildren so that school nutrition programmes are more likely to be implemented in rural areas than urban [24], as observed in Burkina Faso [25].

Recent surveys at national level in Burkina Faso revealed a high prevalence of malnutrition among rural schoolchildren [26]. The aim of the present study was to assess the nutritional status of schoolchildren attending private and public schools covering both the urban and the peri-urban areas of Ouagadougou (Burkina Faso). We hypothesized that undernutrition and micronutrient malnutrition would be widespread, and that public school pupils, particularly in peri-urban areas, would be most affected.

## Methods

### Setting

We conducted between October 2008 and March 2009 a cross-sectional study in 12 public and private schools in Ouagadougou, the capital city of Burkina Faso (West Africa). Ouagadougou is located in the Kadiogo province, Central region. The city and its peri-urban areas were covered in this study which was to serve as baseline for the subsequent implementation and evaluation of the Nutrition Friendly School Initiative of WHO and its partners [27].

### Population and sample

Anaemia was used to estimate sample size, as it is the number one nutritional problem at school age [28]. Based on an estimated prevalence of 40% in this population [26], 350 children were required in each group (intervention and control) in order to allow for detecting a 10% decline in this prevalence after three years of intervention, with 5% alpha error and 80% power. A total number of 770 subjects were selected to allow for refusals and for incomplete data. Only 5^th ^grade classes were included as pupils had to fill-out a self-administered questionnaire. According to the Ministry of primary education, 60% of pupils at this grade can write and read fluently [29]. For practical reasons, all children of the class were invited to take part in the study. As the mean number of pupils per class is around 60 [29], a total of 12 schools was required. The purposive sample of six "intervention" schools in Ouagadougou was selected with the Ministry of primary education according to specific criteria: committed school staff; public and private schools; urban and peri-urban schools; confessional and non-confessional schools; and schools with a complete primary level of six grades, with at least 40% of girls. The intervention schools included 4 public schools (one in a peri-urban area) and 2 private schools (one confessional), all located in different city neighbourhoods. These six schools were then matched with 6 control schools on the basis of size, location (urban/peri-urban), and type (private/public; confessional/non confessional).

### Anthropometric measurements

To assess the nutritional status of pupils, we measured weights and heights according to standard procedures described by WHO [30]. Weight was measured to the nearest 0.1 kg with an electronic scale (SECA 803) with children wearing only light clothing and without shoes. Weight was recorded twice and the mean value was used in the analyses. If the difference between the two measures exceeded 0.2 kg, the child was weighed again. The scale was checked for accuracy with standard weights after about every 200 measures. Individual height was measured with a wooden stadiometer placed on a flat surface. The subject stood on the basal part of the device with feet together (without shoes). The shoulders, the buttocks and the heels had to touch the vertical measuring board. The children standing with their eyes in the Frankfort horizontal plane, the height was measured to the nearest 0.1 cm and recorded twice. Similarly, when the difference between the two measures was higher than 0.5 cm, a third measure was taken and the mean of the two closest values was used in the analyses. Computed Z-scores of Body Mass Index for age (BMIAZ) and height for age (HAZ) were then used to assess thinness/overweight/obesity and stunting, respectively, using the WHO new reference values for school boys and girls [31]. Stunting was defined as HAZ <-2.0, thinness as BMIAZ <-2.0, overweight as BMIAZ >1.0 and obesity as BMIAZ >2.0 [32].

### Biological variables

Haemoglobin (Hb) concentration to assess anaemia was measured in all children with the HemoCue^® ^system (HemoCue, Angelholm, Sweden). The technique is recommended by WHO for field surveys because of its comparability with the cyanmethemoglobin method [33]. One drop of capillary blood is carefully collected at the tip of the middle finger with a lancet. The first two drops are discarded and the third one is used to fill the microcuvette, which is then placed in the cuvette holder of the device (HemoCue Hb 201^+^). The displayed Hb value is then recorded [34]. When the displayed value was lower or equal to 7 g/dl, a second measure was performed and the mean value was recorded for analyses. Age-specfic criteria were used to identify anaemic children: Hb < 11.5 g/dl for children between 7 and 11 years of age, and Hb < 12 g/dl for those aged 12 - 14 years [33].

Vitamin A status was assessed in a random subsample of 208 children (half boys) because of the high cost of the assay. We collected 10 ml of venous blood. After centrifugation at the National Public Health Laboratory (NPHL) of Burkina Faso, the serum samples were analysed in duplicate for retinol with High Performance Liquid Chromatography (HPLC) at the Analytical chemistry laboratory of University of Ouagadougou. The laboratory belongs to a network for quality control of retinol determinations. Low serum retinol indicating vitamin A deficiency (VAD) was defined as < 0.7 μmol/l [35].

Thyroid palpation was performed on all children as a means of assessing iodine deficiency, as described and recommended by WHO to detect goitre in school children [36]. The palpation was performed by a trained medical student in his last year at the Medical School of University of Ouagadougou. The simplified method of grading goitre in three categories was used [36].

### Statistical analyses

Data were processed and analysed with SPSS.17 software (SPSS, Inc., Chicago IL). To ensure data quality, data of 30% of the records were entered twice. Chi square and independent t-test were used for proportions and mean comparisons between groups. All the statistical tests in this study were considered significant at *P *< 0.05.

### Ethical considerations

The study was approved by the research ethics committee of the Faculty of Medicine of University of Montreal and the ethics committee of the Ministry of Health of Burkina Faso. The study's objectives and procedures were explained during meetings held in each school. Informed consent forms were given to the children for their parents to sign and were collected one week later. Children whose parents did not accept undertook other activities with the teachers during data collection. The children themselves also had to agree (orally) to take part in the study, and none refused.

## Results

### Socio-demographic characteristics

**Figure 1:**
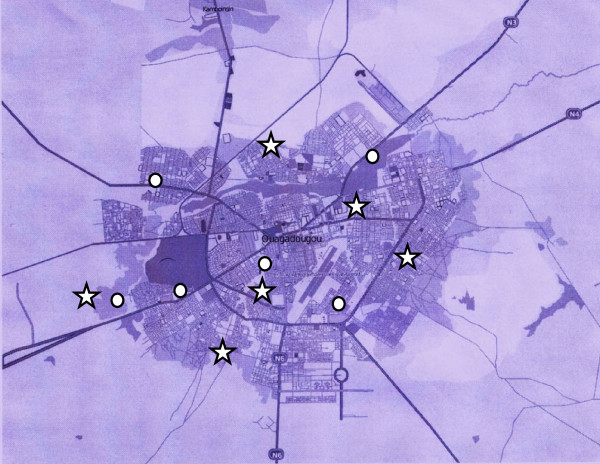
**Map of Ouagadougou locating the 12 schools included in the study**. Star - Intervention schools; Circle - Control schools

Eight public and 4 private schools were included in the study. Their location is shown in Figure 1. As shown in the figure, the selected schools were from various areas throughout the entire city, and two of the eight public schools were located in peri-urban areas. A total of 935 children were invited to participate in the study and 806 parents (86.2%) gave their consent. Since seven subjects were missing at the time of data collection, 799 children were finally included (85.5% response rate). The age ranged from 7 to18 years. We only retained data for the subjects aged between 7 and 14 years (784 subjects) because of the impact of puberty on body measurements. We chose 14 years as cut-off as puberty is likely delayed as frequently observed in developing countries [37,38]. In a longitudinal study in Senegal, mean age at menarche among adolescents (12-17 years old) was 17.2 y, 16.5y and 15.6 y for those who were significantly, mildly, or non-stunted during preschool years, respectively [39]. We also excluded from the analyses pupils whose date of birth was not known (135 subjects). Hence 649 subjects in the total sample and 173 subjects in the sub-sample with complete data were retained for the analyses.

As shown in table 1 the sample included more girls (52.4%) than boys (47.6%). Mean age was 11.5 ± 1.2 years, with 11 years as the mode (34.5%). A total of 457 pupils (70.4%) were in public schools while 192 (29.6%) were in private schools. Peri-urban school children represented 16.3% of the total. The sex ratio was the same in the subsample and in the whole sample. Similarly the proportion of pupils attending public/private (70/30%) and urban/peri-urban (84/16%) schools was roughly the same in the global and the subsample.

**Table 1:** Sociodemographic characteristics of study children

	Total sample (N = 649)				**Sub-sample (N = 173)**^†^			
				
Sociodemographics	*Boys (%)*	*Girls (%)*	*Total (%)*	p**	*Boys (%)*	*Girls (%)*	*Total*	p**
			
School type								
Public	219 (47.9)	238 (52.1)	457 (70.4)		60 (49.6)	61 (50.4)	121 (69.9)	
				*0.808*				*0.518*
Private	90 (46.9)	102 (53.1)	192 (29.6)		23 (44.2)	29 (55.8)	52 (30.1)	
School location								
Urban area	259 (47.7)	284 (52.3)	543 (83.7)		69 (47.6)	76 (52.4)	145 (83.8)	
				*0.921*				*0.815*
Peri-urban area	50 (47.2)	56 (52.8)	106 (16.3)		14 (50.0)	14 (50.0)	28 (16.2)	
Age (years)								
Mean^++^	11.5 ± 1.2	11.5 ± 1.2	11.5 ± 1.2	0.780*	11.6 ± 1.3	11.6 ± 1.3	11.6 ± 1.3	0.919*
7-9	31 (49.2)	32 (50.8)	63 (9.7)		10 (58.8)	7 (41.2)	17 (9.8)	
10-12	241 (47.5)	266 (52.5)	507 (78.1)	*0.959*	59 (46.5)	68 (53.5)	127 (73.4)	*0.631*
13-14	37 (46.8)	42 (53.2)	79 (12.2)		14 (48.3)	15 (51.7)	29 (16.8)	
**Total**	309 (47.6)	340 (52.4)	649 (100)		83 (48.0)	90 (52.0)	173 (100)	

^++ ^Mean age ± SD

* Independent t-test

** χ^2 ^test

**^† ^**208 children (half boys) were randomly selected to assess vitamin A status. Because of the impact of puberty and age on body measurements, we excluded subjects older than 14 years and those whose date of birth was not known. Then 173 subjects in the sub-sample with complete data were retained for the analyses.

### Prevalence of malnutrition

**Figure 2:**
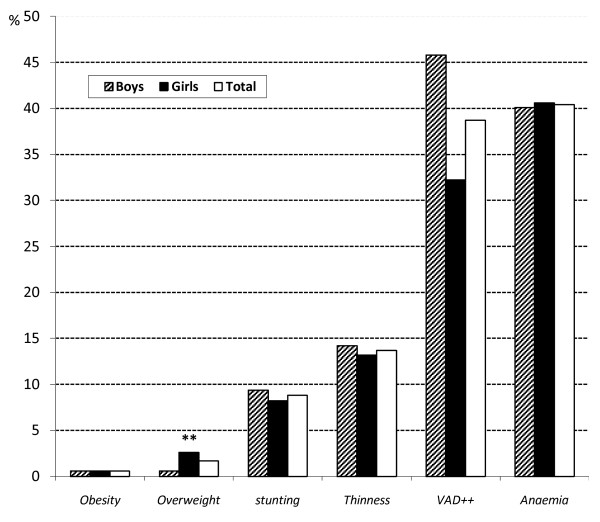
**Prevalence of overall and specific malnutrition indicators in schoolchildren in Ouagadougou, Burkina Faso (N = 649)**. ** p = 0.049 between boys and girls (χ^2 ^test) **^++ ^**N = 173

Figure 2 depicts micronutrient and overall malnutrition rates in the total sample and in the subsample of schoolchildren. Micronutrient malnutrition, namely anaemia and vitamin A deficiency, were highly prevalent, 40.4% and 38.7%, respectively. Stratifying by sex showed that anaemia affected a same proportion of girls and boys in the sample. While 32.2% of girls and 45.8% of boys had vitamin A deficiency, the difference was not significant. No case of goitre was detected with the palpation method.

Regarding overall malnutrition, the stunting rate was 8.8%, 8.2% of girls and 9.4% of boys (non-significant p = 0.605). Thinness affected 13.7% of schoolchildren and there was no significant difference between boys (14.2%) and girls (13.2%). There were only four cases of obesity (two boys and two girls), but it was noted that overweight was slightly higher (1.7%) with a significant difference (p < 0.05) between boys (0.6%) and girls (2.6%). Only one child out of the 77 under 10 years of age was underweight (results not shown).

Table 2 gives the number of malnutrition signs in the subjects. While 43% were within acceptable measures of nutritional well-being, 57% of the children had at least one sign of malnutrition, including 14.6% who presented with two or three indicators of malnutrition. Table 2 also provides details on concurrent deficiencies affecting children according to sex. The combination of anaemia and VAD (20.2%) was the most widespread followed by thinness and anaemia (6%). Only stunting combined with thinness showed a significant difference (p < 0.05) between boys and girls (1.9% and 0.3% respectively).

**Table 2:** Malnutrition signs in schoolchildren in Ouagadougou

	Percentage (Frequency)			
		
Number of signs (N = 649)	Boys	Girls	Total	P value*
**Zero (0)**	**42.1 (130)**	**43.8 (149)**	**43.0 (279)**	
**One (1) or more**	**57.9 (179)**	**56.2 (191)**	**57.0 (370)**	*0.652*
				
**Two (2)**	**14.2 (44)**	**12.1 (41)**	**13.1 (85)**	*0.411*
Stunting + Thinness	1.9 (6)	0.3 (1)	1.1 (7)	*0.042*
Stunting + Anaemia	3.2 (10)	3.5 (12)	3.4 (22)	*0.837*
Thinness + Anaemia	6.5 (20)	5.3 (18)	5.9 (38)	*0.523*
Stunting + VAD**^††^**	3.6 (3)	5.6 (5)	4.6 (8)	*0.544*
Thinness + VAD**^††^**	3.6 (3)	2.2 (2)	2.9 (5)	*0.585*
Anaemia + VAD**^††^**	24.1 (20)	16.7(15)	20.2 (35)	*0.224*
				
**Three (3)**	**1.9 (6)**	**1.2 (4)**	**1.5 (10)**	*0.429*
Stunting + Thinness + Anaemia	1.0 (3)	0.0 (0)	0,5 (3)	*0.069*
Stunting + VAD + Anaemia**^††^**	1.2 (1)	4.4 (4)	2.9 (5)	*0.204*
Thinness + Anaemia + VAD**^††^**	2.4 (2)	0.0 (0)	1.2 (2)	*0.139*

**^†† ^**For multi-deficiencies including VAD, N = 173

* χ^2 ^test

Table 3 displays malnutrition rate by age. The 13-14 year-old group was the most affected by thinness (20.3%) and anaemia (45.6%), followed by those of 7-9 y (14.3%) and 10-12 y (12.6%) for thinness and those of 10-12 y (40.8%) and 7-9 y (30.2%) for anaemia. The children aged 10-12 years old were the most affected with VAD (43.3%). However, the age differences in proportions of micronutrient malnutrition were not statistically significant.

**Table 3:** Nutritional status of schoolchildren in Ouagadougou according to age

		*Nutritional status (%)*				
		
*Age (years)*	N	Overweight/Obesity	Thinness	Stunting	Anaemia	Vitamin A deficiency ^††^
7-9	63	4 (6.3)	9 (14.3)	0 (0.0)	19 (30.2)	3 (17.6)
10-12	507	11 (2.2)	64 (12.6)	42 (8.3)	207 (40.8)	55 (43.3)
13-14	79	0 (0.0)	16 (20.3)	15 (19.0)	36 (45.6)	9 (31.0)
P value*		*0.039*	*0.184*	*< 0.001*	*0.160*	*0.081*

**Total**	649	15 (2.3)	89 (13.7)	57 (8.8)	262 (40.4)	67 (38.7)

**^†† ^**On sub-sample only (N = 173)

* χ^2 ^test

The differences in the prevalence of stunting across age groups were statistically significant (p < 0.001), and so were the differences of overweight/obesity (p < 0.05). Youngest children (7-9 y) did not present with stunting while the older children (13-14 y) were the most affected group with 19.0% prevalence, followed by 10-12 y group (8.3%). Conversely, while older children did not present with overweight/obesity their younger peers (7-9 y) were more affected (6.3%) followed by the 10-12 y group with 2.2% prevalence.

When comparing the nutritional status of the children according to school type (table 4), it appears that pupils attending public schools were significantly more affected by micronutrient malnutrition than those of private schools (p = 0.001 and p < 0.001 for anaemia and VAD, respectively). Stunting was more frequent in public schools (9.6%) compared to private schools (6.8%), although the difference was not significant. Interestingly, overweight/obesity was significantly (p = 0.009) more prevalent in private schools (4.7%) compared to public schools (1.3%) whereas the prevalence of thinness was almost the same in the two school types (13% and 14% respectively for private and public schools).

**Table 4:** Malnutrition of schoolchildren in Ouagadougou according to school characteristics

		*Nutritional status (%)*				
		
*School characteristics*	N	Overweight/obesity	Thinness	Stunting	Anaemia	Vitamin A deficiency^††^
**School type**						
Public schools	457	6 (1.3)	64 (14.0)	44 (9.6)	204 (44.6)	64 (52.9)
Private schools	192	9 (4.7)	25 (13.0)	13 (6.8)	58 (30.2)	3 (5.8)
P value*		*0.009*	*0.740*	*0.241*	*0.001*	*< 0.001*
**School location**						
Urban schools	543	15 (2.8)	68 (12.5)	40 (7.4)	221 (40.7)	52 (35.9)
Peri-urban schools	106	0 (0.0)	21 (19.8)	17 (16.0)	41 (38.7)	15 (53.6)
P value*		*0.083*	*0.046*	*0.004*	*0.698*	*0.078*
**Total**	649	15 (2.3)	89 (13.7)	57 (8.8)	262 (40.4)	67 (38.7)

**^†† ^**On sub-sample only (N = 173)

*χ^2 ^test

As depicted in table 4 stunting (16.0%) and thinness (19.8%) were significantly higher in peri-urban than urban schools (p = 0.004 and p < 0.05 respectively). VAD tended to be more widespread and overweight/obesity lower in the peri-urban schools than in the urban schools.

## Discussion

The present study showed that malnutrition, whether undernutrition or micronutrient deficiencies, was highly prevalent at school age in urban areas. Almost 60% of the children examined had at least one sign of malnutrition and roughly 15% had at least two such indicators. Of particular concern, more than 40% of the subjects were anaemic and roughly the same percentage were vitamin A deficient. We verified that there was no significant difference in the prevalence of micronutrient malnutrition in the retained subjects compared to the 135 children excluded because their birth date was unknown (p = 0.133 and p = 0.183 for VAD and anaemia, respectively: data not shown). These high rates in children (mean age 11.5 ± 1.2 years) are similar to those of the national study of schoolchildren in Burkina Faso, whose mean age was 9.7 ± 5.8 years [26]. In this study where rural schoolchildren were predominant, 40.5% were vitamin A deficient and 43.7% were anaemic. Similarly, in the baseline study of the red palm oil project in selected primary schools of two zones of Burkina Faso (out of the Central region, where Ouagadougou is located), more than 40% of the children were vitamin A deficient [40]. A high prevalence of micronutrient malnutrition at school age is not uncommon in developing countries [12]. In northern Ethiopia, the prevalence of VAD was 51.1% in a study conducted in 1997 in 824 pupils aged 6-9 years [41]. In a report on six African and two Asian countries, 40.2% of children aged 7-11 years and 54.4% of those aged 12-14 years were anaemic [12]. A similar increasing trend of anaemia with age is observed in the current study (table 3).

We also found that 13.7% of the children were thin, which is higher than the 8% prevalence previously reported for Burkina Faso schoolchildren outside the capital city of Ouagadougou [26]. Thinness, or wasting, usually describes acute malnutrition. Our study was conducted between late 2008 and early 2009, that is, during the global economic and food crisis that hit developing countries so hard [42] and which was responsible for reduced access to food particularly among vulnerable populations [43]. We observed that several schoolchildren stayed at school during lunchtime, but did not have pocket money to buy any street food, or did not have enough to eat an adequate meal. This may have played a role in the observed prevalence of thinness in schoolchildren of Ouagadougou. Notwithstanding, this level is far lower than that reported by the Partnership for Child Development (PCD) in schoolchildren of developing countries ten years ago [44].

The prevalence of stunting in our study was lower than in the recent national study of schoolchildren in Burkina Faso (8.8% *vs *12%) [26], as well as in a nationwide survey in Chad (18.7%) in a sample of schoolchildren aged between 6 and 15 years [45]. Stunting is an indicator of chronic malnutrition, and at school age, it may reflect malnutrition during the first years of life [1]. Growth deficit tends to accumulate with age and particularly in boys, as observed in our study and in other studies of school-children in developing countries [44]. The higher rate of stunting among older children, depicting an increasing vulnerability with age may also reflect some improvement of food and health conditions over recent years since most of the growth deficit or catch-up takes place before the age of 24 months [46]. Except for overweight/obesity and anaemia, a higher proportion of boys than girls showed signs of malnutrition, as previously reported for stunting and wasting [44], and for VAD [41]. A metanalysis of data from 16 demographic and health surveys conducted in 10 sub-Saharan countries [23] revealed that boys were more stunted than girls, and speculated on the role of cultural factors or natural selection [23].

Although malnutrition still appears as a priority problem, overweight/obesity should not be overlooked right at school age, as we detected a higher prevalence trend in the youngest group of children. At variance with our study, a much lower prevalence of obesity was reported in 2001 (0.26% vs 0.60%) in an adolescent population of Ouagadougou (mean age 13.8 y) [47]. However, both studies are consistent in the significantly higher prevalence of overweight observed in girls compared with boys (Figure 2).

It was clearly apparent in our study that private school-children enjoyed a better nutritional status than those attending public schools, with anaemia and VAD significantly higher in the latter (30% *vs *45% and 6% *vs *53% respectively). However, it is of note that overweight/obesity was also significantly higher in private than public schools, which is in accordance with previous reports in other developing country schoolchildren [48]. Socio-economic disparities likely underlie these differences [48]. We did not examine the socio-economic conditions of the individual children, but mere differences in school registration fees are convincing: US $ 60 in private schools compared with only US $ 4 in public schools. Nevertheless, it is surprising that thinness was as common in private as in public schools in our study (13.0% and 14.0%, respectively). There is no obvious explanation for this high rate of thinness even in private school children.

As could be expected, stunting and thinness were significantly higher in peri-urban than urban schools, and VAD also tended to be higher in the former than latter schools (table 4). Poverty and low maternal education are among the determinants of child malnutrition [49]. It is also known that the prevalence of malnutrition is higher in rural than urban areas, particularly stunting [9], which reflects poor socio-economic status as the community level. It is therefore not surprising to find a higher percentage of malnourished children in peri-urban areas, where people are poorer, and where schools also draw their pupils from the surrounding villages. Anaemia was observed in roughly the same proportion of urban and peri-urban schoolchildren (around 40%). This confirms that anaemia is the most widespread malnutrition problem in schoolchildren in developing countries [28].

While iron deficiency is the main factor of anaemia [50], it is not the only one, and infection plays a major role [15,51], notably malaria and hookworms in African school-children [14]. In Ouagadougou, for instance, the prevalence of malaria (41.4%) tended to be the highest in children aged 5-14 years, who were also at the highest risk of infection compared to infants and adults [52]. Other micronutrient deficiencies may also be involved in the aetiology of anaemia [53]. The "top three" micronutrient deficiencies are iron deficiency, VAD and Iodine Deficiency Disorders (IDD) [28]. The high prevalence of anaemia could be a great threat for school-children, particularly since it was combined with VAD in one out of five children (20.2%) in our study. Indeed iron deficiency and VAD are interrelated [54-56]. In contrast, we detected no goitre using the palpation method recommended by WHO [36], which likely reflects the effectiveness of the salt iodization strategy of the past several decades [57].

To our knowledge, this nutrition study is the first of its kind among city schoolchildren of West Africa. Although the schools were not randomly selected, they represent a broad array of features: public and private, confessional and non-confessional, as well as urban and peri-urban schools. Furthermore, sample size was large enough and in a narrow age-range. However, because of these study features, the results cannot be extrapolated.

School nutrition and feeding programs are usually directed at rural areas [25]. Furthermore, under-five children are the priority target group for strategies and actions to fight malnutrition. There is an urgent need to address nutrition problems among schoolchildren in developing countries, without neglecting urban areas, considering that malnutrition can impair their performance while in school and their productivity later on in life [58].

## Conclusion

Based on our findings, it appears that undernutrition and micronutrient deficiencies are prominent even in urban schoolchildren. Overweight/obesity is still uncommon but it is appearing in private schools and amongst younger children. It may be concluded that the nutrition transition characterized by shifts in dietary habits and lifestyles with resulting increases in the prevalence of obesity and co-morbidity is still in its early stages in the area of the study. The high prevalence of VAD and anaemia and their frequent combination [59] should be of concern and underlines the compelling need for corrective and preventive measures in urban schools, which should no longer be neglected in favour of rural areas.

## Competing interests

The authors declare that they have no competing interests.

## Authors' contributions

CD, HD and OR designed the study. CD collected and analysed the field data under the supervision of HD and the co-supervision of OR. CD drafted the paper, HD and OR reviewed the draft and made some changes. All authors read and approved the final manuscript.
